# The short-term effect of nitrogen and phosphorus fertilizers on cold resistance in *Urtica cannabina* based on transcriptomics and metabolomics analysis

**DOI:** 10.3389/fpls.2025.1598628

**Published:** 2025-05-14

**Authors:** Siqi Liu, Xiaoxue Zhang, Guorui Zhang, Jinmei Zhao, Xiaoqing Zhang

**Affiliations:** ^1^ Institute of Grassland Research, Chinese Academy of Agricultural Science, Hohhot, China; ^2^ College of Life Sciences, Northwest A &F University, Yangling, Shan Xi, China; ^3^ Northern Agriculture and Livestock Husbandry Technology Innovation Center, Chinese Academy of Agricultural Science, Hohhot, China

**Keywords:** *Urtica cannabina*, nitrogen fertilizer, phosphate fertilizer, cold resistance, overwintering period

## Abstract

**Introduction:**

Freezing injury in winter is a major abiotic stress that significantly limits plant growth and survival. While nitrogen and phosphorus fertilizers have been demonstrated to alleviate the impact of freezing injury in various plant species, their role of fertilizers in the cold tolerance of *Urtica* spp. is still unknown.

**Methods:**

This study investigated the effects of fertilizers on the cold resistance of *U. cannabina* by comprehensively analyzing the physiological and biochemical indices, transcriptome, and metabolome of the *U. cannabina* under applications of 150 kg nitrogen ha-1 (N) and 90 kg phosphorus ha-1 (P), using “no fertilizer” (CK) as the control.

**Results:**

The results showed that applying nitrogen and phosphorus fertilizers reduced the malondialdehyde concentration and had much higher superoxide dismutase activity and soluble sugar and proline concentrations. Transcriptomics and metabolomics analysis revealed that applying nitrogen and phosphorus fertilizers tended to involve several critical regulatory pathways in the biosynthesis of secondary metabolites, flavonoid biosynthesis, and phenylpropanoid biosynthesis pathways. Concretely speaking, these fertilizers can affect the biosynthesis of naringenin, pinobanksin 3-acetate, galangin, and p-Coumaroyl shikimic acid and the expression of related genes to regulate the cold tolerance of *U. cannabina*. Moreover, through using weighted correlation network analysis (WGCNA), 4210 genes in response to nitrogen fertilizer and 5975 genes in response to phosphorus fertilizer, positively correlating with key metabolites, were identified. Several genes encoding enzymes including glucan endo-1,3-beta-glucosidase, pectinesterase, trehalase, hydroquinone glucosyltransferase, monodehydroascorbate reductase, tyrosine aminotransferase, and peroxidase were verified to be hub genes involved in the cold-stress response of *U. cannabina*.

**Discussion:**

Overall, these findings have laid a theoretical foundation for the highly efficient utilization of nitrogen and phosphorus in *U. cannabina* and provide novel insights into the regulatory network of *U. cannabina* in response to cold-temperature stress.

## 1 Introduction

*Urtica cannabina* L., a perennial herb of *Urtica* spp., has strong adaptability and high nutritional value. As a kind of food and Chinese herbal medicine, *U. cannabina* is rich in terpenoids, flavonoids, coumarins, sugars, and other bioactive substances (Li et al., 2009; Wang et al., 2016; Tarasevičienė et al., 2023), with good antioxidant, antibacterial, and hypoglycemic biological functions (Wang et al., 2018; Long et al., 2020). Additionally, *U. cannabina* has high amounts of protein, vitamins, minerals, and unsaturated fatty acids, which are rich in nutrients and can be used as a non-conventional feed (Kregiel et al., 2018; Zhang et al., 2024). For this reason, it is of practical significance to increase the feed source of livestock (especially of high-protein forage) and alleviate the contradiction between forage and livestock by artificial cultivation of high-yield and high-quality *U. cannabina*.

In recent years, climate change-driven temperature fluctuations have led to a significant threat to perennial plant cultivation and agricultural production (Wang et al., 2025). When the temperature drops to 0°C, plants may suffer serious damage, including blocked physiological activities, slowed root growth, cell damage, freezing injury, and cold damage (Jan and Andrabi, 2009). Nitrogen and phosphorus are essential macronutrients for plant growth and overwintering, serving as the main constituent of proteins, amino acids, and nucleic acids such as adenosine triphosphate (ATP) (Da Silva and Williams, 2001). Safi et al. (2021) described the relationship between reactive oxygen species (ROS) levels and nitrogen demand, where nitrogen deficiency led to elevated ROS concentrations and reduced antioxidant activity, indicating that adequate nitrogen supply is crucial for establishing cellular redox balance. Raghothama (1999) and Plaxton and Tran (2011) reported that phosphorus mainly participates in energy metabolism; adequate phosphorus supply helps maintain the normal function of antioxidant enzymes, thereby supporting essential life activities and stress responses in plants. Many recent studies have demonstrated that the application of nitrogen or phosphorus fertilizers significantly alleviate the impact of freezing injury and increase the winter survival rate of alfalfa (Sun et al., 2022; Wang et al., 2022). Sun et al. (2022) and Wang et al. (2022) reported that application of 44 or 100 kg ha^-1^ phosphorus fertilizer significantly increases the overwintering rate of alfalfa. Hu (2006) found that nitrogen fertilizer (0.24 g kg^-1^ soil) significantly increases the soluble sugar concentration in leaves of *U. dioica* (a genus of *Urtica* spp.), while phosphorus fertilizer has no significant effect. The possible mechanism was that the phosphate induced the biosynthesis of n-acetyl-l-phenylalanine, l-serine, lactose, and isocitrate, thus improving alfalfa’s tolerance to cold (Wang et al., 2023). However, the effect of nitrogen or phosphorus fertilizer on the cold resistance of *U. cannabina* during natural cold-temperature dormancy remains unclear.

In the present study, the cultivated *U. cannabina* was exposed to CK, N, and P treatments for 150 days. Superoxide dismutase activity and the concentrations of malondialdehyde, soluble sugar, and proline were measured. In addition, transcriptomic and metabolomic analyzes were performed between the N vs. CK and P vs. CK in cold temperatures. The integrated transcriptome-metabolome analysis allowed a deeper understanding of the response to applications of nitrogen and phosphorus in terms of *U. cannabina*’s cold tolerance. The aim is to provide fine insights into the differential expression of genes and different metabolites related to nitrogen and phosphorus utilization in *U. cannabina* during cold stress, which could be used to improve the tolerance and yield of *U. cannabina*.

## 2 Materials and methods

### 2.1 Study site

The experiment was conducted in the Agro-pastoral Experiment Station (N 40°34′, E 111°45′; altitude 1,050 m a.s.l.) in Inner Mongolia, China. Here, the climate is semi-arid continental with a mean annual precipitation of 400 mm and a yearly average temperature of 5.6°C (Wang et al., 2022). Generally, extremely low and high temperatures range from -17.6°C to 30.6°C from January to July during the growing seasons (**Supplementary Table S1**). The accumulated temperature (≥ 10°C) is above 2700°C, and the frost-free period is about 150 days from April to September. The soil type is meadow soil and sandy loam. The soil within the 20 cm layer has organic matter of 1.32%, total nitrogen of 0.08%, available nitrogen of 88.26 mg kg^−1^, available phosphorus of 5.47 mg kg^−1^, available potassium of 88.00 mg kg^−1^, and pH of 8.65.

### 2.2 Field experiment design

The variety of *U. cannabina* used in this study was provided by the Grassland Research Institute of the Chinese Academy of Agricultural Sciences (Hohhot, China). The experiment began in early August of 2022 at the Agro-pastoral Experiment Station. Seeds were planted manually. Treatments were arranged in a randomized complete block design with a split-plot arrangement at three fertilizer levels. Based on the preliminary experiment, the three fertilizer treatments were (i) no fertilizer (CK), (ii) 150 kg ha^-1^ of nitrogen (N), and (iii) 90 kg ha^-1^ of phosphorus (P), respectively. Each treatment field was randomly assigned to three replicated plots (3.2 m × 6.3 m). The seeding rate was 2.60 kg ha^-1^ at a soil depth of less than 0.5 cm and 30 cm row spacing. No other fertilizer was supplied throughout the experiment, and the plants were watered and weeded as required during the plant-growing period.

### 2.3 Sample preparation and physiological indices analysis

The root crowns of *U. cannabina* were collected in mid-January 2023. Thirty crowns served as biological replicates. The root crowns were rinsed with clean water, wiped with absorbent paper, cut into 1–2 cm pieces, and quickly placed into frozen tubes, stored in liquid nitrogen, and then stored at -80°C for further analysis.

The activity of superoxide dismutase (SOD) was determined by the nitrogen blue tetrazole colorimetric method (Cai and Gao, 2020); the concentration of malonaldehyde (MDA) was determined by the thiobarbiturate method; the concentration of proline (Pro) was determined by ninhydrin colorimetry, and the concentration of soluble sugar (SS) by the anthrone colorimetry method. The above indices were determined using the SINABESTBIO kits (SINABESTBIO, Shanghai, China). The data of those physiological indices were statistically analyzed using a one-way analysis of variance (ANOVA) of SPSS software (version 25.0), and multiple comparisons — *i.e.*, Tukey’s test — were performed at a probability (*P*) value < 0.05.

### 2.4 Metabolite profiling and data analysis

The extraction, detection, and quantitative analysis of metabolites in the samples were performed by Wuhan Metware Biotechnology Co., Ltd. (www.metware.cn). The root samples were freeze-dried and crushed in a blender (MM 400, Retsch). Approximately 50 mg of the powder was dissolved in 1200 μL of 70% methanol, vortexed every 30 min for 30 s, and stored overnight at 4°C. Subsequently, the extract was centrifuged at 11,304 g for 3 min, and the supernatant was filtered through a microporous filter membrane (0.22 μm pore size) into a sample vial. The sample extracts were analyzed using an UPLC-ESI-MS/MS system (UPLC, ExionLC™ AD, https://sciex.com.cn/) and a Tandem mass spectrometry system (https://sciex.com.cn/). The analytical conditions were as follows: UPLC column, Agilent SB-C18 (1.8 µm, 2.1 mm × 100 mm); the mobile phase was consisted of solvent A, pure water with 0.1% formic acid, and solvent B, acetonitrile with 0.1% formic acid. Sample measurements were performed with a gradient program that employed the starting conditions of 95% A, and 5% B. Within 9 min, a linear gradient to 5% A, 95% B was programmed, and a composition of 5% A, 95% B was kept for 1 min. Subsequently, a composition of 95% A, 5.0% B was adjusted within 1.1 min and kept for 2.9 min. The flow velocity was 0.35 mL per minute; the column oven was set to 40°C; the injection volume was 2 μL. The effluent was alternatively connected to an ESI-triple quadrupole-linear ion trap (QTRAP)-MS.

The ESI source operation parameters were as follows: source temperature 550°C; ion spray voltage (IS) 5500 V (positive ion mode)/-4500 V (negative ion mode); ion source gas I (GSI), gas II (GSII), curtain gas (CUR) were set at 50, 60, and 25 psi, respectively; the collision-activated dissociation (CAD) was high. QQQ scans were acquired as MRM experiments with collision gas (nitrogen) set to medium. DP (declustering potential) and CE (collision energy) for individual MRM transitions was done with further DP and CE optimization. A specific set of MRM transitions was monitored for each period according to the metabolites eluted. The orthogonal partial least squares discriminant analysis (OPLS-DA) and principal component analysis (PCA) were carried out on all the samples to identify the putative biomarkers after data normalization. Finally, the multiple variations of Fold-Change |(FC)| ≥ 2 or ≤ 0.5 and Variable Importance in Projection (VIP) ≥ 1 were identified as the differentially expressed metabolites (DEMs). The differential metabolites were annotated using the Kyoto Encyclopedia of Genes and Genomes (KEGG) database, followed by enrichment pathway analysis.

### 2.5 Transcriptome sequencing and data analysis

Total RNA was extracted from each sample using an RNA extraction kit (Beijing Tiangen Biotechnology, China), according to the manufacturer’s instructions. The qualified RNA was used to establish a cDNA library. The prepared cDNA libraries were sequenced on the Illumina Novaseq 6000 system by Wuhan Metware. The raw RNA-Seq data were submitted to NCBI (www.ncbi.nlm.nih.gov/bioproject/PRJNA1139937). After obtaining the raw sequences, Fastp software (version 0.23.2) was used to remove adapter sequences; paired reads were discarded if the N content in any read exceeded 10% of the total bases or if low-quality bases (Q ≤ 20) constituted more than 50% of the read, to obtain clean reads for use in subsequent analyzes. Transcriptome assembly was performed using Trinity software (version 2.13.2) using both the cleaned Illumina short reads and Nanopore long reads by implementing the long-reads flag. The integrity of the assembled transcriptome was evaluated by performing Benchmarking Universal Single-Copy Orthologs (BUSCO) software (version 5.4.3) analysis using the embryophyta_odb10 dataset. Then using TransDecoder software (version 5.3.0) to identify candidate protein coding regions with default parameters.The fragments per kilobase of transcript per million fragments mapped (FPKM) were calculate by RESM software (version 1.3.1). The differentially expressed genes between groups (DEGs) was analyze by the DESeq software (version 1.22.2), and the resulting p-values were adjusted using Benjamini & Hochberg’s method to control the false discovery rate (FDR). The final DEGs were defined as by parameters of FDR < 0.05 and |log2FC| ≥ 1. The enrichment analysis was performed based on the hypergeometric test.

### 2.6 Weighted gene co-expression network analysis

The WGCNA (version 1.71) package in R was used to generate the co-expression network module between 22 key metabolites, biochemical characteristics, and genes via the dynamic treecut method (Langfelder and Horvath, 2008). The weighting factor β satisfied a correlation coefficient close to 0.63. In this study, β =18 was chosen as the weighting factor. The modules with minModule size = 50 and merge cut height = 0.25 were used as standards to merge modules with a similarity of 0.75. Transcription factors (TFs) analysis was performed using iTAK software (version 1.7a). Cytoscape software (version 3.10.1) was used to visualize the gene network of the top 50 hub genes within the module.

### 2.7 Real-time quantitative PCR

To validate the transcriptome data, eight DEGs were selected for qRT-PCR validation of the RNA-seq data using three technical replicates per sample. See **Supplementary Table S2** for the list of primers.

## 3 Results

### 3.1 Physiological responses of *U. cannabina* in the different treatments

**Figure 1:**
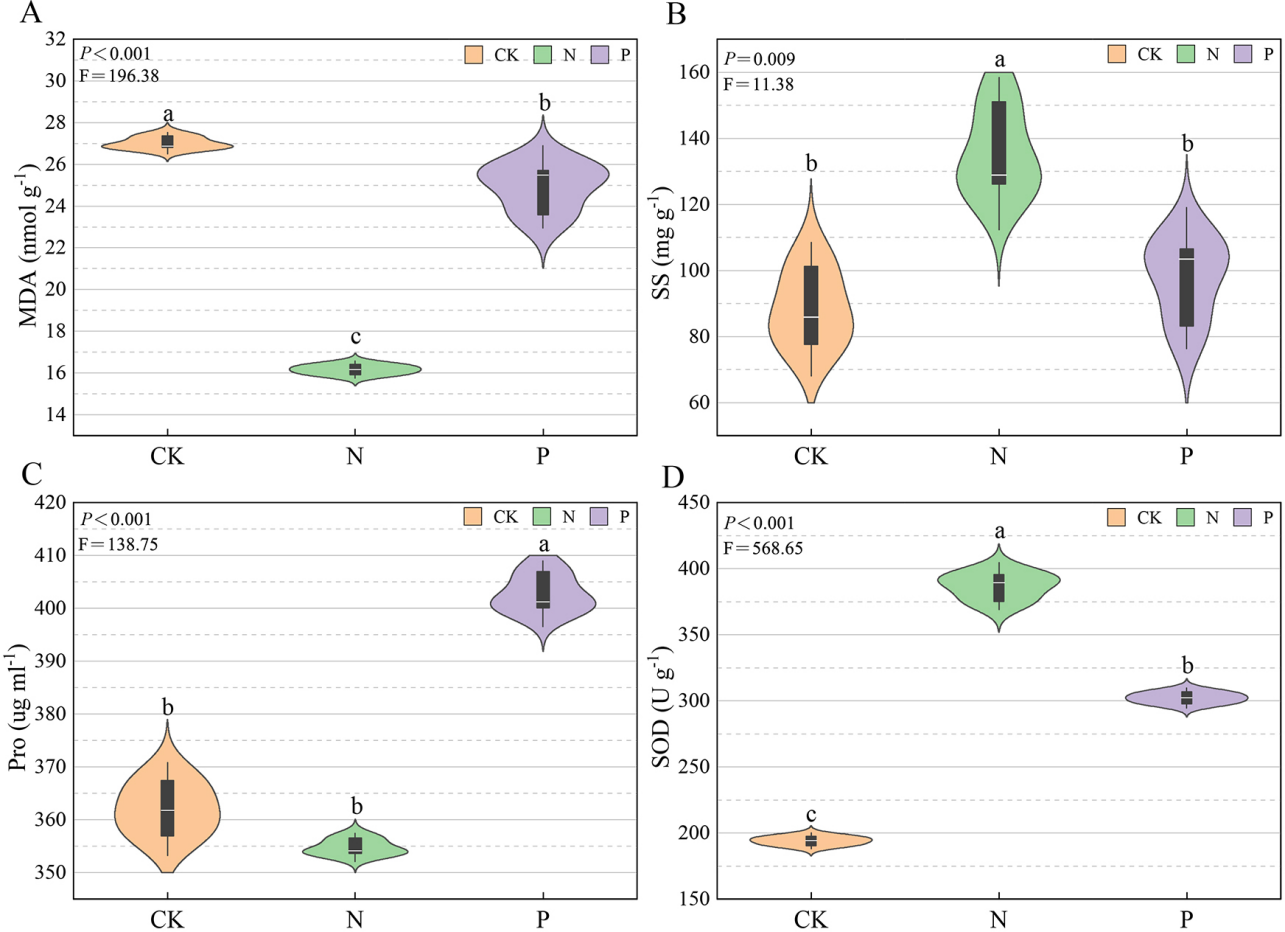
The physiological indices of *U. cannabina* in the different treatments. **(A)** Malondialdehyde (MDA) concentration. **(B)** Soluble sugar (SS) concentration. **(C)** Proline (Pro) concentration. **(D)** Superoxide dismutase (SOD) activity.

As shown in **Figure 1**, the highest MDA concentration was in the CK treatment, the medium level was in the P treatment, and the lowest value was in the N treatment (*P* < 0.05). The SS concentration was significantly (*P* < 0.05) higher in the N than the CK treatment, and there was no significant difference between the P and CK treatments. The Pro concentration was significantly higher (*P* < 0.05) in the P than in the CK treatment, and there was no significant difference between the N and CK treatments. The N treatment had the highest activity of SOD, followed by the P treatment, and the lowest in the CK treatment (*P* < 0.05).

### 3.2 Metabolomic analysis in the different treatments

**Figure 2:**
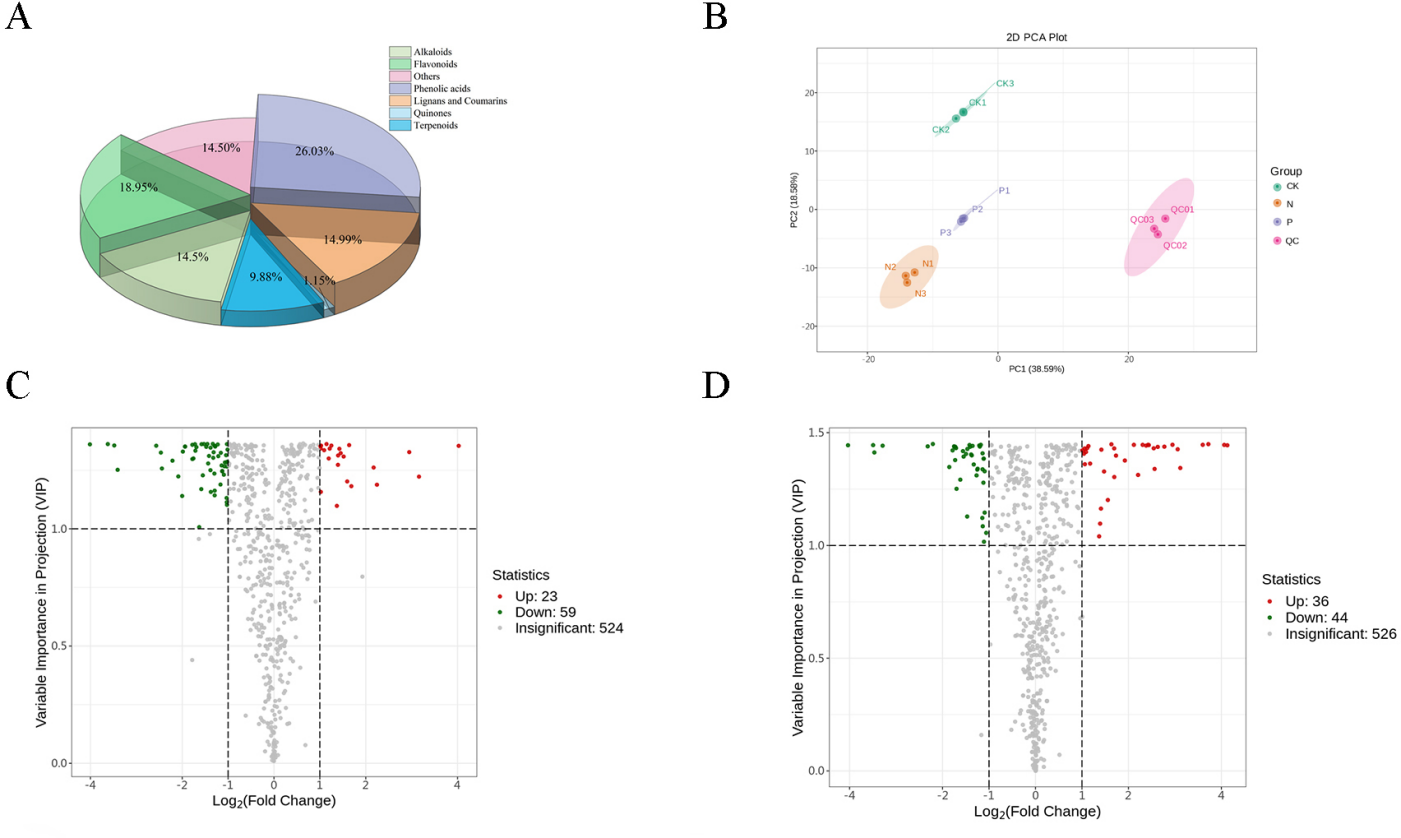
Overview and analysis of metabolites. **(A)** Total number of differentially expressed metabolites classes. **(B)** PCA of metabolome data in the three treatments (CK, N, and P). **(C)** Differentially expressed metabolites of the CK and N treatments. **(D)** Differentially expressed metabolites of the CK and P treatments.

**Figure 3:**
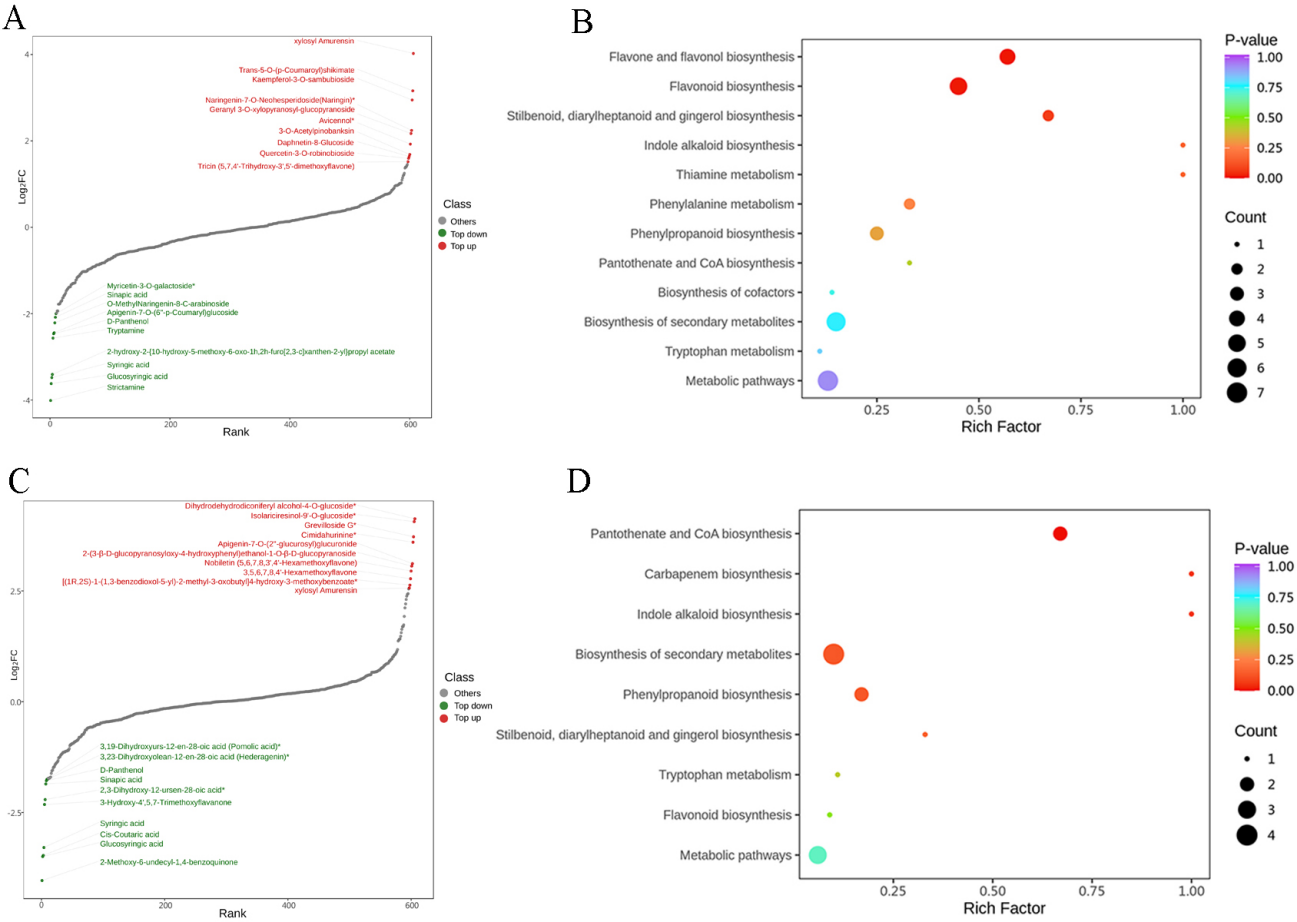
**(A)** Top 20 differentially expressed metabolites in the CK and N treatments. **(B)** KEGG pathway analysis of the CK and N treatments. The horizontal coordinate indicates the Rich factor of each pathway (Rich factor was calculated as the ratio of the number of differentially expressed genes annotated in a pathway to the number of all genes annotated in this pathway), and the vertical coordinate is the pathway’s name. **(C)** Top 20 differentially expressed metabolites in the CK and P treatments. **(D)** KEGG pathway analysis of the CK and P treatments.

A total of 607 metabolites (**Supplementary Table S3**) were identified across samples, including a large proportion of phenolic acids, flavonoids, lignans, coumarins, and alkaloids (**Figure 2A**). From the PCA score plots (**Figure 2B**), the metabolites were clearly spread among the CK, N, and P treatments. Further, the distribution of different DEMs can be easily visualized by volcano plots (**Figures 2C, D**). In comparing the CK and N treatments, a total of 82 DEMs (**Supplementary Table S4**) were identified, of which strictamine, glucosyringic acid, syringic acid, and 2-{10-hydroxymo-5-methoxykoto-6-[2m-3] xanthen-2-yl} propyl acetate were significantly downregulated, while xylosyl amurensin, trans-5-o-(p-coumaroyl) shikimate, and kaempferol-3-o-sambubioside were significantly upregulated in the N treatment (**Figure 3A**). Further KEGG analysis showed that the flavonoid biosynthesis, flavone and flavonol biosynthesis, and the stilbenoid, diarylheptanoid and gingerol biosynthesis pathways were significantly enriched in the N treatment (**Figure 3B**). In comparing the CK and P treatments, a total of 80 DEMs (**Supplementary Table S5**) were identified, of which syringic acid, cis-coutaric acid, glucosyringic acid, and 2-methoxy-6-undecyl-1,4-benzoquinone were significantly downregulated, while dihydrodehydrodiconiferyl alcohol-4-o-glucoside, isolariciresinol-9’-o-glucoside, grevilloside G and cimidahurinine were significantly upregulated in the P treatment (**Figure 3C**). The biosynthesis of secondary metabolites, phenylpropanoid biosynthesis, and pantothenate and CoA biosynthesis pathways were significantly enriched in the P treatment (**Figure 3D**).

### 3.3 Transcriptomic analysis in the different treatments

**Figure 4:**
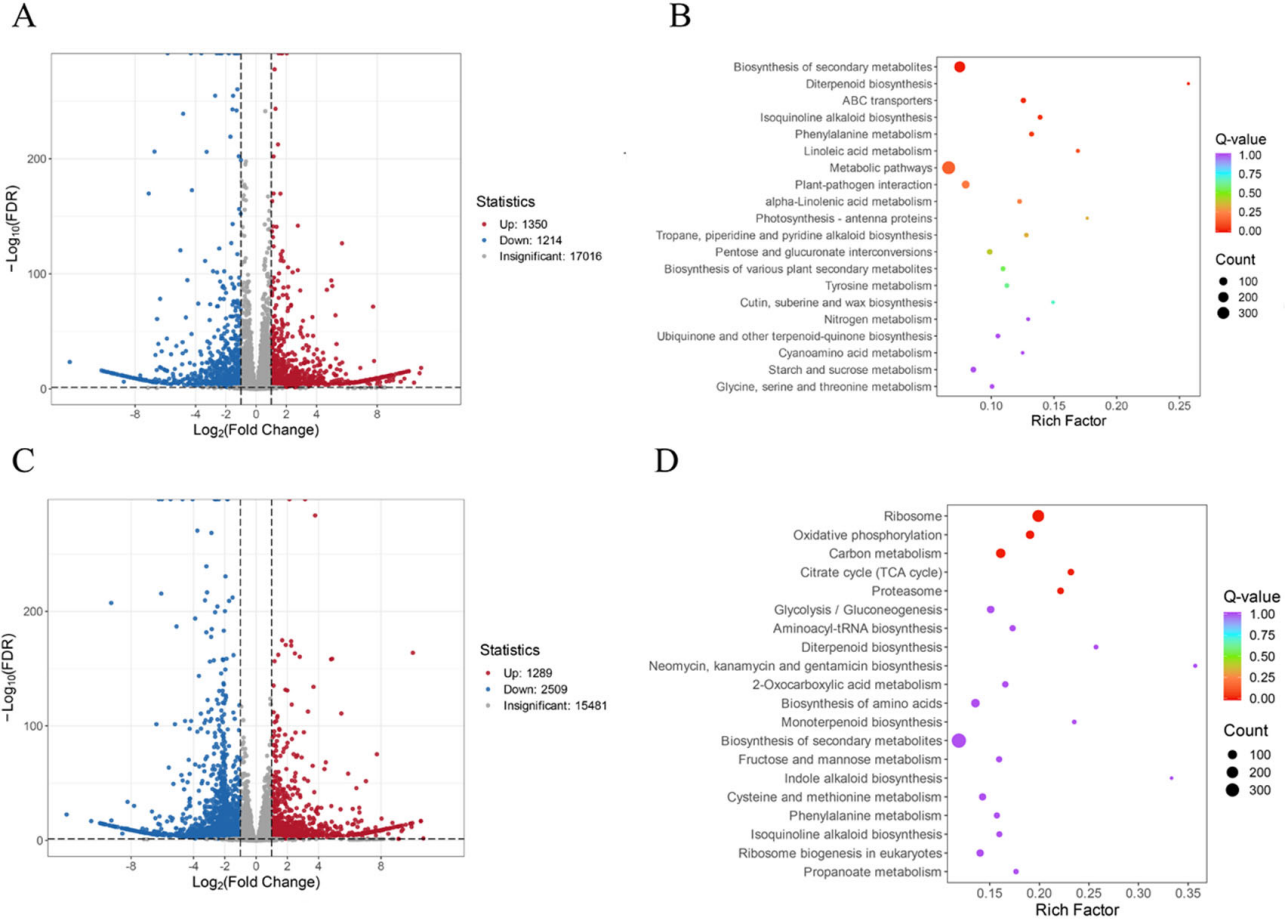
Differentially expressed genes analysis. **(A)** Differentially expressed genes of the CK and N treatments. **(B)** KEGG pathway analysis of the CK and N treatments. **(C)** Differentially expressed genes of the CK and P treatments. **(D)** KEGG pathway analysis of the CK and P treatments.

After removing low-quality reads, 89.68 Gb clean data were obtained, with an error rate of less than or equal to 0.03%. The values of Q20, Q30, and GC were above 97.48%, 92.98%, and 49.42%, respectively (**Supplementary Table S6**). The sequencing quality was good and could be used for further DEG analysis. In comparing the CK and N treatments, a total of 2,564 DEGs were identified, including 1,350 upregulated genes and 1,214 downregulated genes in the N treatment (**Figure 4A**, **Supplementary Tables S7**, **S8**). Based on the KEGG gene annotation analysis of the DEGs, it was found that the biosynthesis of secondary metabolites, ABC transporters, phenylalanine metabolism, and isoquinoline alkaloid biosynthesis pathways were significantly enriched in the N treatment (**Figure 4B**). Compared to the CK, a total of 3,798 DEGs were identified in the P treatments, including 1,289 upregulated genes and 2,509 downregulated genes (**Figure 4C**, **Supplementary Tables S7**, **S8**), which were significantly enriched in the oxidative phosphorylation, ribosome, citrate cycle, carbon metabolism, and proteasome pathways (**Figure 4D**).

### 3.4 Identification of key genes and modules based on WGCNA analysis

**Figure 5:**
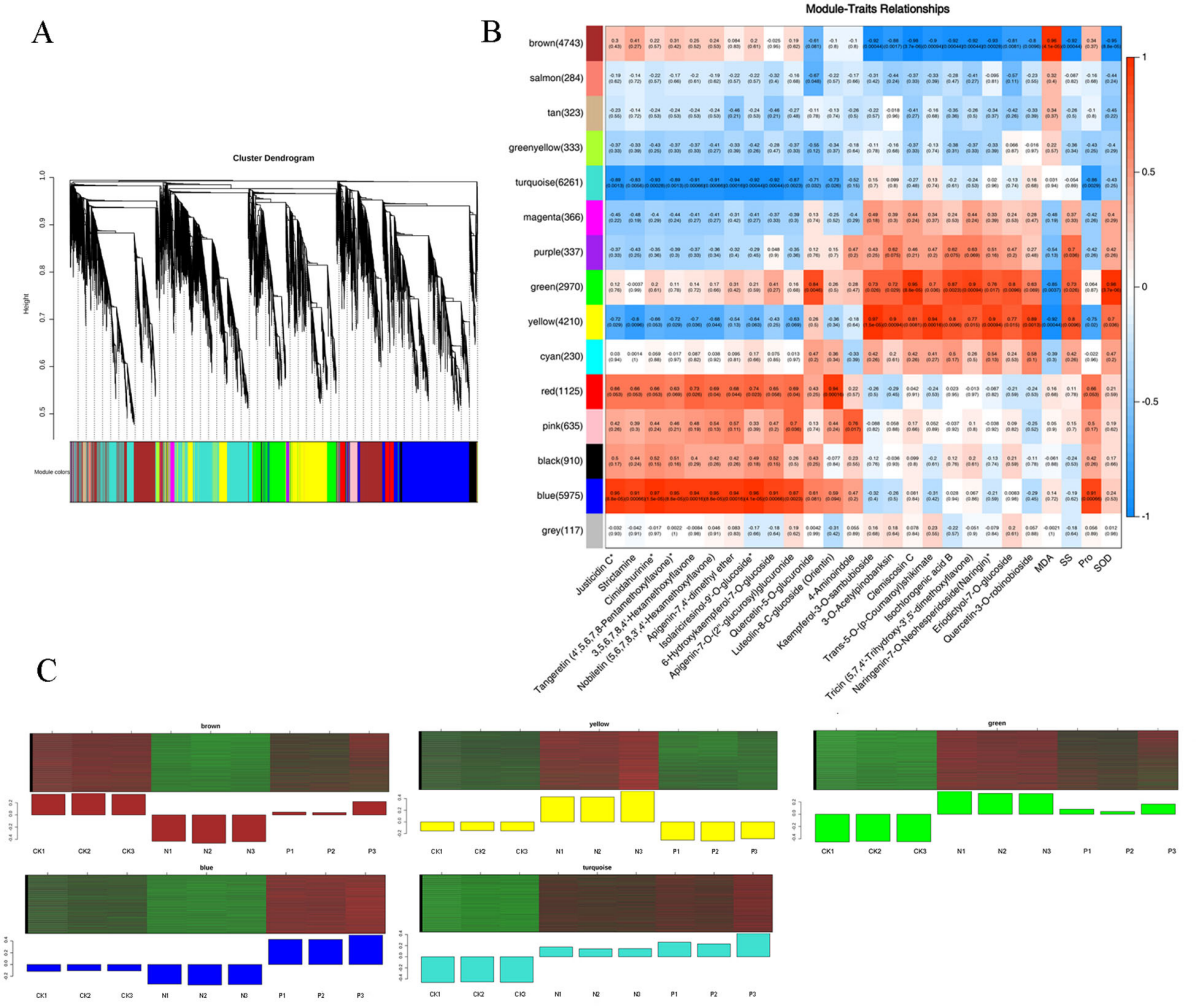
Identification of hub genes and modules responsive to cold stress in *U. cannabina* by weighted gene co-expression network analysis. **(A)** The hierarchical cluster tree shows 17 modules of coexpressed genes. **(B)** Module-trait associations based on Pearson correlations. Each row corresponds to a specific module eigengene, and a column to a trait. The values in each cell represent the correlation coefficients (r) and the *p*-values (in parentheses) of the module-trait association. **(C)** Eigengenes expression profiles of the brown, turquoise, green, yellow, and blue module.

**Figure 6:**
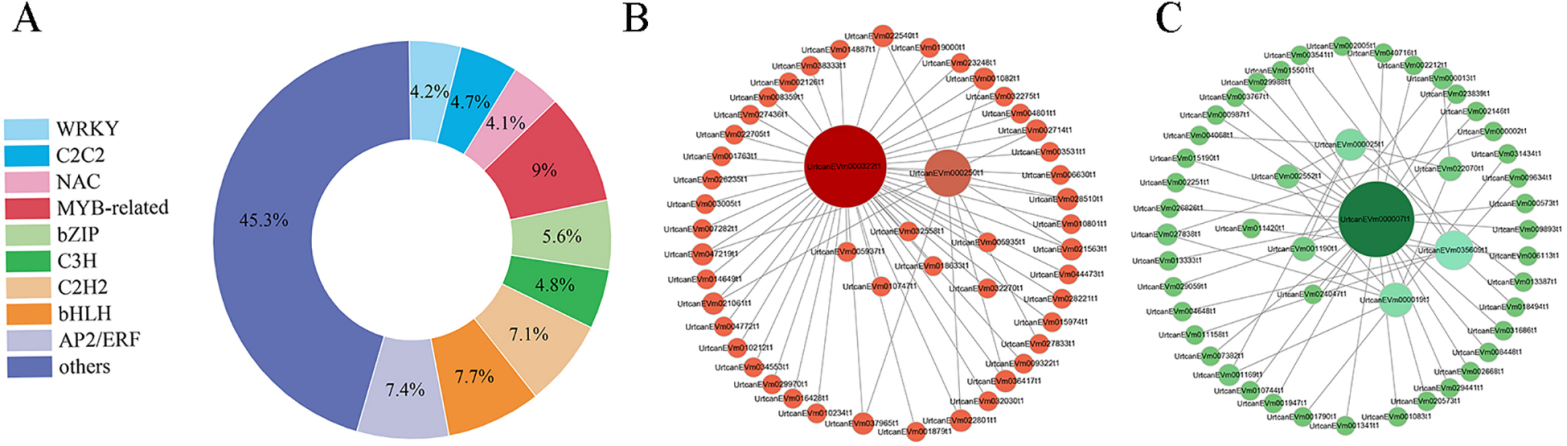
**(A)** The family distribution of transcription factors. Visualization of the key co-expression network yellow module **(B)** and the blue **(C)** module by Cytoscape.

Based on similar expression patterns, a total of 15 co-expression modules were identified from the different samples (**Figure 5A**). The correlation heat map between modules and different samples is shown in **Figure 5B**. Considering the modules significantly related to gene expression patterns in the module-trait relationships analysis, five special modules (brown, turquoise, green, yellow, and blue) were found, and the diagram of gene expression patterns was plotted (**Figure 5C**). The 6261 genes in the turquoise module and 5975 genes in the blue module displayed opposite expression patterns in the CK and P treatments. The yellow module contained 4210 genes, and the brown module contained 4743 genes with opposite expression profiles in the CK and P treatments. The 2970 genes in the green module were positively associated with the N and P treatments, respectively. A total of 887 differentially expressed TFs from 5 special modules were divided into 47 categories (**Supplementary Table S9**). These TFs were classified into 10 prominent families, with the following distribution: 66 AP2/ERF, 43 C3H, 68 bHLH, 80 MYB-related, 36 NAC, 42 C2C2, 63 C2H2, 50 bZIP, 37 WRKY, and 402 Others (**Figure 6A**). The TFs families AP2/ERF, MYB-related, C2H2, and bHLH were found to be the most active participants in *U. cannabina*’s response to cold stress. At the same time, two gene co-expression networks were constructed using the top 50 hub genes with high connectivity from the two highly correlated key modules (**Figures 6B, C**). Notably, those genes in **Table 1** encoded some of the important proteins involved in abiotic stress processes, including glucan endo-1,3-beta-glucosidase, pectinesterase, alpha-trehalase, and hydroquinone glucosyltransferase in the yellow module, and monodehydroascorbate reductase, arginine decarboxylase, tyrosine aminotransferase, adenylate kinase, and peroxidase in the blue module.

**Table 1:** The selected candidate hub-gene based on module-trait relationship analysis.

Modlue	Identification	KWithin	KEGG gene function notation
Yellow	UrtcanEVm005937t1	902.23	glucan endo-1,3-beta-glucosidase
	UrtcanEVm001585t1	727.20	alpha-trehalase
	UrtcanEVm017018t1	645.07	tyrosine aminotransferase
	UrtcanEVm018633t1	935.71	pectinesterase
	UrtcanEVm005935t1	928.95	hydroquinone glucosyltransferase
	UrtcanEVm002318t1	485.79	arginine decarboxylase
Blue	UrtcanEVm022070t1	1134.90	peroxidase
	UrtcanEVm000440t1	645.54	adenylate kinase
	UrtcanEVm035609t1	1303.73	monodehydroascorbate reductase
	UrtcanEVm010596t1	1127.95	tyrosine aminotransferase

### 3.5 Integration of flavonoid biosynthesis and phenylpropanoid biosynthesis pathways

**Figure 7:**
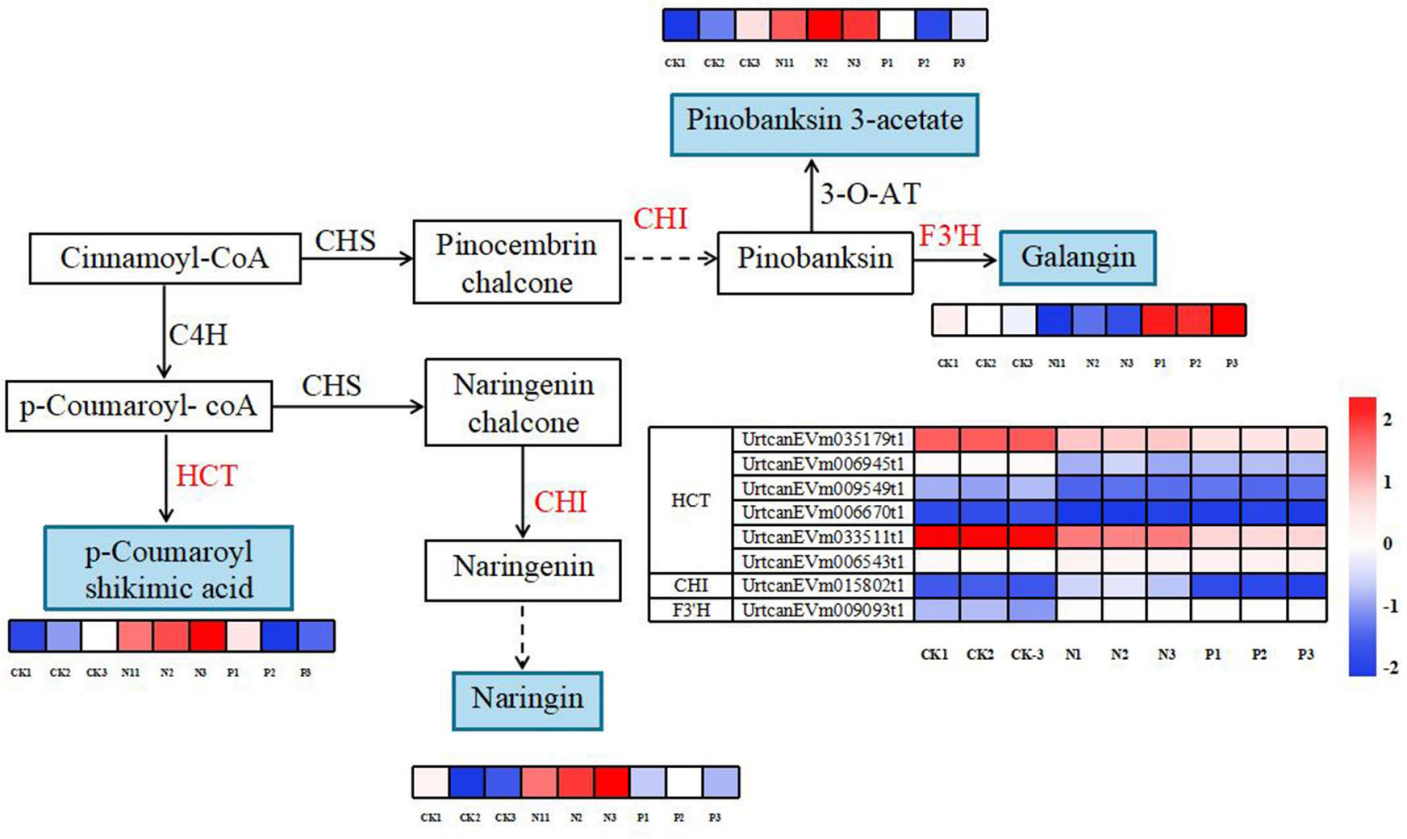
Diagram of flavonoid and phenylpropanoid biosynthesis pathways with their related differentially expressed genes and differentially expressed metabolites. Note: The solid line indicates the metabolic reactions in only one step. The dashed line presents more than one step of the metabolic reaction.

A total of four DEMs and eight DEGs were enriched in flavonoid biosynthesis and phenylpropanoid biosynthesis pathways (**Figure 7**). Compared to the CK treatment, the accumulation of p-coumaroyl shikimic acid, naringin, and pinobanksin 3-acetate was significantly higher in the N treatment, and galangin accumulated was significantly higher in the P treatment. Additionally, the expression patterns of DEGs involved in both metabolic pathways are shown in **Figure 7**. In particular, the genes of *UrtcanEVm035179t1* and *UrtcanEVm033511t1* encoding HCT were upregulated in the CK, N and P treatment. The remaining genes were unchanged or only slightly downregulated.

### 3.6 Validation of DEGs by qRT-PCR

**Figure 8:**
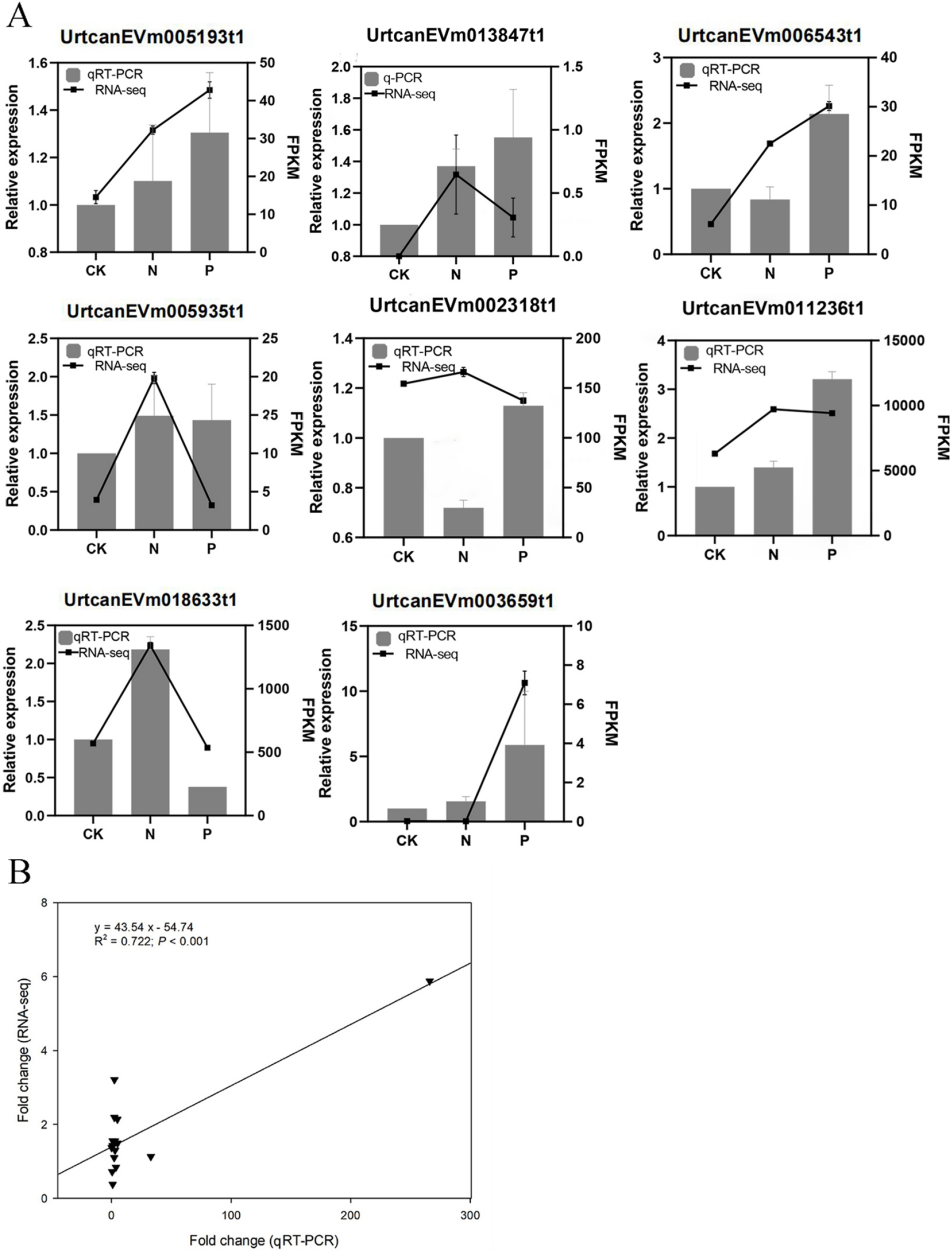
**(A)** The results of RT-qPCR with eight genes. **(B)** Relationship between qRT-PCR and RNA-seq.

A total of eight DEGs that play key roles in flavonoid biosynthesis and phenylpropanoid biosynthesis pathways, and some hub genes involved in gene and metabolite related networks, were selected for qRT-PCR to verify the accuracy of RNA sequencing. The results showed that the qRT-PCR data were consistent with the transcriptomic data, displaying the same expression pattern (R^2^ = 0.722, *P* < 0.001; **Figure 8**).

## 4 Discussion

### 4.1 Physiological responses to nitrogen and phosphorus fertilizers applied to *U. cannabina*

It is well known that the physiological characteristics of roots can reflect plant growth, absorption capacity, and cold-temperature adaptability. The MDA is an important indicator to assess cell membrane integrity and oxidative damage caused by lipid peroxidation (Lee et al., 2004). Liu et al. (2010) suggested that applying nitrogen (240 kg ha^-1^) could significantly reduce the concentration of MDA in *Lolium multiflorum* during the overwintering period, thereby reducing the damage caused by cold temperature to the membrane system. Their finding agrees with the results of the present study that MDA concentration in the N and P treatments separately significantly decreased by 40.13% and 7.74% compared to the CK treatment probably because nitrogen and phosphorus play key roles in various biochemical, antioxidant enzymatic, and metabolic activities, as well as serve as the structural components of many plant compounds, and increase plant growth and vigor (Singh et al., 2015; Ma et al., 2022). The study’s results not only illustrate that the N and P treatments alleviate the damage to cell membranes but also increase the activities of enzymatic *e.g.* SOD and non-enzymatic *e.g.* pro and SS) antioxidants. Generally, the enhanced activities of SOD confer on plants the capability to scavenge reactive oxygen species (ROS) and resist cold stress (Mittler, 2002; Scandalios, 2005). This finding is supported by Chen et al. (2021), who found that the application of phosphate (300 kg ha^-1^) significantly increased the SOD activity of alfalfa roots at -20°C, improving their antioxidant capacity. The role of Pro and SS is variable. They act as radical scavengers in plants under stress as well as an osmotic agent (Sanchez et al., 2008; Hayat et al., 2012). Liu et al. (2011) found that when natural cold stress occurred in turf-type bluegrass, nitrogen fertilizer significantly improved the cold resistance of this plant by increasing the SS concentration in its leaves. Ihtisham et al. (2023) found that nitrogen and phosphorus significantly increased Pro concentration in perennial ryegrass and mitigated cold stress-provoked adversities compared to the control conditions. The elevated antioxidant activities in the N and P treatments correspond to better ROS scavenging ability. They are conducive to *U. cannabina’s* recovery from cold stress and repair of the damage caused by cold stress. The above results demonstrate that applying nitrogen and phosphorus fertilizers can improve *U. cannabina*’s adaptability to cold stress.

### 4.2 Adaptation strategies of *U. cannabina* to nitrogen and phosphorus fertilizer application

The results of the current study clearly show that the primary role of the N and P treatments in improving cold resistance in *U. cannabina* was achieved through its impact on the biosynthesis of secondary metabolites, flavonoid biosynthesis, and phenylpropanoid biosynthesis pathways. Some flavonoids, such as naringin, pinobanksin 3-acetate, and galangin (3,5,7-trihydroxyflavone), can help plants withstand cold temperatures by reducing the incidence of lipid peroxidation and oxidative damage and scavenging free radicals (Jagetia et al., 2004; Seyoum et al., 2006; Chen et al., 2023). Studies have reported that higher naringin accumulated in cold-tolerant peaches than in cold-sensitive peaches by cold stress (Li et al., 2023) and the accumulation of galangin can protect against oxidative damage (Ekambaram et al., 2023). This flavanone accumulation is part of the flavonoid biosynthesis pathway, activated to enhance the plant’s cold tolerance. The result of the current study indicates that the N and P treatments enhanced the accumulation of naringin and galangin in their roots. The results also show that compared with the CK, the N and P treatments had higher antioxidant defenses and mitigating oxidative stress expressed by inducing the accumulation of pinobanksin 3-acetate. Specifically, naringin undergoes further modifications, including specific enzymes that introduce hydroxyl and acetyl groups to the naringin molecule, leading to the accumulation of pinobanksin 3-acetate (Falcone Ferreyra et al., 2012). The research presented here is supported by the reports of Sharma et al. (2019) and Jiang et al. (2024) on the accumulation of pinobanksin 3-acetate contributing to a plant’s antioxidant defenses and overall stress tolerance.

Moreover, the results reveal that the phenylpropanoid biosynthesis pathways produce many secondary metabolites, including flavonoids, phenolic acids, and other aromatic metabolites (Vogt, 2010). The production of phenolic acids, such as p-coumaroyl shikimic acid, is closely associated with the coumarin biosynthesis pathway within the phenylpropanoid biosynthesis pathways. Prior studies reported that p-coumaroyl shikimic acid helps plants survive and reproduce at cold temperatures by increasing antioxidant capacity, reducing cell membrane damage, and regulating the expression of cold-responsive genes (Thomashow, 2010; Di Ferdinando et al., 2012). The results of this study reveal that the N treatments can promote the accumulation of p-coumaroyl shikimic acid by affecting the coumarin biosynthesis pathway, indirectly enhancing the cold tolerance of *U. cannabina*. These findings are partially consistent with those of Zeng et al. (2024), who stated that the levels of HCT (hydroxycinnamoyltransferase) could potentially inhibit the coumarin biosynthesis pathway when nitrogen was applied to *Lithocarpus polystachyus.*

In brief, the N and P treatments modify biochemical mechanisms in *U. cannabina* to shield it from the detrimental impacts of cold stress. Previous researchers found that the application of nitrogen and phosphorus fertilizers boosted the accumulation of phenolic compounds in stinging nettle (Biesiada et al., 2009; Radman et al., 2015), and the strong association between polyphenols and abiotic stress tolerance was an excellent predictor of the extent of plant tolerance (Hodaei et al., 2018). In the current study, naringin, pinobanksin 3-acetate, p-coumaroyl shikimic acid, and galangin (3,5,7-trihydroxyflavone) were the most abundant flavonoids and phenolic acids detected in the N and P treatments, which coincides with a dual protective effect as antioxidants guard against oxidative damage induced by the stress. This not only had a clear motivation but was also supported by the accumulation of antioxidant enzymes, MDA, SS, and Pro in *U. cannabina* in the N and P treatments, which play crucial physiological and biochemical roles in plant cells, particularly in mitigating cold stress.

### 4.3 Discovery of candidate regulatory genes in *U. cannabina* response to cold stress

Certain proteins in plant cells that provide protective functions against external environmental stress are regulated by specific TFs. These TFs families play positive or negative regulatory roles in the plant’s response to abiotic stress. The results of this study indicate that most of the differentially expressed TFs belong to the AP2/ERF-ERF, bHLH, MYB-related, and C2H2families. Previous studies have shown that the CBF/DREB subfamily of the AP2/ERF superfamily plays a critical role in cold stress tolerance (Liu et al., 2022). When the CsbHLH18 transcription factor is overexpressed in citrus, the corresponding antioxidant enzymes become more active, effectively reducing the accumulation of reactive oxygen species (ROS) and enhancing the cold tolerance of the transgenic plants (Geng, 2018). Additionally, Chen et al. (2013) demonstrated that MYB family genes, such as MYB14 and MYB15, enhance cold tolerance in *Arabidopsis* by regulating and suppressing the expression of CBF genes. The SlCZFP1gene in tomatoes has been reported to induce cold-responsive genes and improve cold tolerance (Zhang et al., 2010). Based on these findings, we conclude that these TFs play parallel roles in response to abiotic stress conditions in *U. cannabina*.

By analyzing these hub genes, the genes of *UrtcanEVm017018t1* in the yellow module and *UrtcanEVm010596t1* in the blue module mainly encode tyrosine aminotransferase. Tyrosine aminotransferase plays a role in the metabolic pathways of aromatic amino acids, which are precursors to various secondary metabolites. The secondary metabolites, such as phenolics and flavonoids, can contribute to the plant’s defense mechanisms against cold stress by acting as antioxidants (Dixon and Paiva, 1995; Kaplan et al., 2004). The synthesis and storage of phenolic and flavonoid glycosides are facilitated by various glucosyltransferases, including hydroquinone glucosyltransferase for specific substrates (Andreu et al., 2023; Tak et al., 2023). Therefore, *UrtcanEVm005935t1* (encoding hydroquinone glucosyltransferase) from the yellow module was probably involved in the glycosylation of hydroquinone, leading to the formation of glycosylated compounds, including eriodictyol-7-o-glucoside, kaempferol-3-o-sambubioside, naringenin-7-o-neohesperidoside, and quercetin-3-o-robinobioside in the N treatment. On the other hand, glucan endo-1,3-beta-glucosidase provides soluble sugar to the plant cells mainly by degrading cellulose, which reduces the cytoplasmic osmotic potential and the freezing point, maintaining the stability of the plant cellular structure (Han et al., 2023). The pectin esterase regulates the hardness and flexibility of the plant cell wall by degrading pectin, making the plant cell wall more stable and flexible (Micheli, 2001). In addition, Shen et al. (2020) reported that genes encoding trehalose in upland cotton of KN27–3 type are highly expressed under cold-temperature stress, which promotes the accumulation of soluble sugar and protects cotton from oxidative damage. Specifically, the research presented here is supported by the authors’ earlier works bestowing the N treatment on SS, which may have been related to the three hub genes of *UrtcanEVm005937t1* (encoding glucan endo-1,3-beta-glucosidase), *UrtcanEVm018633t1* (encoding pectinesterase), and *UrtcanEVm001585t1* (encoding alpha-trehalase).

Two genes (*UrtcanEVm022070t1* and *UrtcanEVm035609t1*) encoding oxidoreductase, and one gene (*UrtcanEVm002318t1*) encoding arginine decarboxylase (ADC) were selected in the blue and yellow modules. Oxidoreductases such as peroxidase and monodehydroascorbate reductase mainly protect plant cells from oxidative damage caused by cold stress by regulating plant ROS levels (Mittler, 2002). ADC is one of the key enzymes in the polyamine biosynthesis pathway. It catalyzes the decarboxylation of arginine to produce putrescine, which provides an essential precursor for the synthesis of spermidine and spermine (Kusano et al., 2008). Mitsuya et al. (2009) showed that the growth and survival rate of transgenic rice seedlings overexpressing the ADC gene in cold temperatures increased, enhancing cold stress tolerance. The concentrations of polyamine, Pro, and ascorbate peroxidase were significantly increased in the transgenic crops overexpressing ADC, as well as the expression of CBFs and cold-related genes (Mustafavi et al., 2018). These data, together with a much higher Pro concentration in the P treatment, suggest further evidence of the relationship between the ADC and Pro in response to cold stress, particularly helping in cold stress of *U. cannabina*. Of particular interest was the one gene (*UrtcanEVm000440t1*) predicted to encode adenylate kinase, selected in the blue module, which plays a vital role in plant cell energy homeostasis, mainly by catalyzing the mutual conversion of adenine nucleotides Plaxton and Podestá (2006). The regular supply of energy metabolism enables *U. cannabina* to resist cold-temperature damage continuously. In conclusion, the network formed by the above physiological and biochemical pathways comodulates the freezing tolerance in *U. cannabina*. Here, the short-term physiological and molecular responses to nitrogen and phosphorus fertilizers on cold resistance in *U. cannabina* were clarified clearly. However, whether these findings are applicable to other regions or climates, as well as their long-term impacts on growth, yield, and cold tolerance in *U. cannabina*, should be investigated in future studies to inform strategies for optimizing fertilization application in its cultivation.

## 5 Conclusions

To the authors’ knowledge, this is the first report that provides detailed information on the physiological, transcriptional, and metabolic responses of *U. cannabina* roots to applications of nitrogen and phosphorus fertilizers. Based on these multi-level results, such applications significantly reduce the degree of cell membrane peroxidation of *U. cannabina* roots while strengthening the antioxidant capacity of this plant. The integration of the transcriptomic and metabolomic analyses shows that the application of nitrogen and phosphorus fertilizers can affect the biosynthesis of naringenin, pinobanksin 3-acetate, galanin, and p-coumaroyl shikimic acid and the expression of the hub genes to regulate the cold tolerance of *U. cannabina*.

## Data Availability

The datasets presented in this study can be found in online repositories. The names of the repository/repositories and accession number(s) can be found in the article/**Supplementary Material**.

## Supplementary material

Supplementary Table 1 Precipitation and air temperatures in the Agro-pastoral Experiment Station.

Supplementary Table 2 List of primers used for qRT-PCR analysis of selected genes in *U. cannabina*.

Supplementary Table 3 A list of metabolites detected in the samples.

Supplementary Table 4 The list of changed differential metabolites between the N vs. CK under cold stress in *U. cannabina*.

Supplementary Table 5 The list of changed differential metabolites between the P vs. CK under cold stress in *U. cannabina*.

Supplementary Table 6 Evaluation and statistics of transcriptome sequencing data of *U. cannabina*.

Supplementary Table 7 The table of gene reads count.

Supplementary Table 8 The table of differentially expressed genes.

Supplementary Table 9 The classification table of transcription factors.
